# Risk of major psychiatric disorder after adolescent pregnancy: A nationwide cohort study of 149,870 girls

**DOI:** 10.1111/pcn.70054

**Published:** 2026-03-26

**Authors:** Tien‐Wei Hsu, Jia‐Ru Li, Shih‐Jen Tsai, Ya‐Mei Bai, Tung‐Ping Su, Tzeng‐Ji Chen, Ju‐Wei Hsu, Mu‐Hong Chen, Chih‐Sung Liang

**Affiliations:** ^1^ Department of Psychiatry E‐DA Dachang Hospital, I‐Shou University Kaohsiung Taiwan; ^2^ Department of Psychiatry E‐DA Hospital, I‐Shou University Kaohsiung Taiwan; ^3^ Graduate Institute of Clinical Medicine, College of Medicine Kaohsiung Medical University Kaohsiung Taiwan; ^4^ School of Medicine, College of Medicine I‐Shou University Kaohsiung Taiwan; ^5^ Department of Psychiatry Far Eastern Memorial Hospital New Taipei City Taiwan; ^6^ Department of Psychiatry Taipei Veterans General Hospital Taipei Taiwan; ^7^ Department of Psychiatry, College of Medicine National Yang Ming Chiao Tung University Taipei Taiwan; ^8^ Department of Psychiatry Cheng Hsin General Hospital Taipei Taiwan; ^9^ Department of Family Medicine Taipei Veterans General Hospital Taipei Taiwan; ^10^ Institute of Hospital and Health Care Administration National Yang Ming Chiao Tung University Taipei Taiwan; ^11^ Department of Family Medicine Taipei Veterans General Hospital, Hsinchu Branch Hsinchu Taiwan; ^12^ Department of Psychiatry Tri‐Service General Hospital, National Defense Medical Center Taipei Taiwan; ^13^ Department of Psychiatry, Beitou Branch Tri‐Service General Hospital Taipei Taiwan

**Keywords:** adolescent pregnancy, bipolar disorder, major depressive disorder, major psychiatric disorders, schizophrenia

## Abstract

**Aim:**

Adolescent pregnancy is a global public health concern associated with increased risks of physical health, mental well‐being, and social functioning; however, its potential to increase the long‐term risk of major psychiatric disorder (MPD) remains underexplored.

**Methods:**

Between 1996 and 2013, a cohort of 29,974 girls with adolescent pregnancy without MPD before and 1:4 matched controls based on age, family income, residence location, and individual psychiatric comorbidity were enrolled. A Cox regression model was used, adjusted for potential confounders, to estimate the risk of schizophrenia, bipolar disorder, and major depressive disorder (MDD).

**Results:**

After adjusting for confounding factors, adolescent pregnancy was associated with significantly increased risks of bipolar disorder (hazard ratio 1.44; 95% confidence interval 1.20–1.72) and MDD (1.88; 1.76–2.00), but not schizophrenia (0.94; 0.73–1.21), compared to the control group. These associations persisted after excluding the first 3 years of follow‐up, with elevated risks of bipolar disorder (1.27; 1.05–1.54) and MDD (1.61; 1.50–1.72) among adolescents with pregnancy compared to controls.

**Conclusions:**

These results underscore the importance of implementing long‐term mental health surveillance strategies for young mothers who experience adolescent pregnancy.

Despite a global decline in teenage birth rates,^1^ adolescent pregnancy remains a major global health challenge, with approximately 16 million adolescent girls giving birth each year, particularly in low‐ and middle‐income countries.^2^ Evidence has shown that adolescent pregnancy is linked to a higher risk of maternal anemia, infections, eclampsia and preeclampsia, emergency cesarean sections, and delayed initiation of breastfeeding.^3^ Babies born to teenage mothers are more likely to be premature, have low birth weight, and face an increased risk of respiratory distress syndrome and autism later in life.^3^ Importantly, early pregnancies are also associated with negative social determinants, such as lower economic status, decreased educational opportunities, abuse by family members, and stigmatization.^4^

Mental disorders play an important role in teenage pregnancy. Depression and insecure attachment style have been associated with elevated rates of adolescent pregnancy.^5^, ^6^ Adolescents whose depression onset coincided with sexual initiation had an increased risk of teenage pregnancy.^7^ Additionally, because of higher levels of impulsivity, individuals with attention‐deficit/hyperactivity disorder,^8^, ^9^ substance use disorders,^10^ and bipolar disorders^11^, ^12^ have been linked to greater risk of unplanned pregnancy during adolescence.

Notably, childbirth is a significant trigger for mania and psychosis, with episodes during this period leading to considerable morbidity and mortality.^13^ Females with bipolar disorder are at increased risk of symptom exacerbation during pregnancy or the immediate postpartum period, as indicated by a nearly sevenfold higher risk of admission for a first episode.^14^ Another cohort study reported a substantially higher risk of the first onset of schizophrenia and admission to psychiatric wards following childbirth.^15^ However, these studies have two notable limitations. First, they do not specifically differentiate between adolescent mothers and adult mothers. Second, the follow‐up period is mostly concentrated in the immediate postpartum phase, without providing information on the long‐term risk of mental disorders.

Although some evidence suggests an increased risk of major depressive disorder following adolescent pregnancy,^16^, ^17^ the long‐term risks of major psychiatric disorders (MPDs) remain largely unexplored. To address this gap, we conducted a nationwide longitudinal study using the Taiwan National Health Insurance Research Database (NHIRD) to examine the subsequent risks of schizophrenia, bipolar disorder, and major depressive disorder among adolescent mothers. Importantly, parental history of MPDs was incorporated as a covariate in the adjustment models, given its strong association with the risk of subsequent psychiatric disorders. We hypothesized that adolescent pregnancy would be associated with increased long‐term risks of developing MPDs.

## Methods

### Data source

Following the formal application for a research project, the National Health Research Institute conducts an audit and provides the Taiwan NHIRD for scientific and research purposes. The Longitudinal Health Insurance Database of the NHIRD, which includes all medical records from 1996 to 2013, provided the data for the current study analysis. The dates of clinical visits, diagnoses, treatments, and prescriptions for 3,000,000 insured individuals, selected at random from the approximately 28,000,000 Taiwanese population, were included in this dataset. The International Classification of Diseases, Ninth Revision, Clinical Modification (ICD‐9‐CM) served as the source of the diagnostic codes used. The NHIRD has been extensively used in many epidemiologic studies in Taiwan.^18^, ^19^, ^20^, ^21^

### Participants

Female adolescents aged 12–19 years who experienced adolescent pregnancy between 1 January 1996, and 31 December 2013, and had no prior history of any MPDs – namely, schizophrenia, bipolar disorder, or major depressive disorder – were included in the present study. Pregnancy was determined using pregnancy‐related procedure codes, such as obstetric ultrasound evaluation, vaginal birth, cesarean section, and abortion procedures, or pregnancy‐related diagnostic codes (ICD‐9‐CM codes: 63, 64, 65, 66, 67*x* [*x* = 0–7], 779.6, V22, V23, V24, V27). In this study, the exposure was defined as evidence of adolescent pregnancy in claims data (pregnancy *per se*), regardless of subsequent pregnancy outcome (live birth, spontaneous abortion, or induced abortion). The index date was the first date the pregnancy was determined, and the same date was applied to the controls. All time‐to‐event analyses used time since the pregnancy index date as the underlying time scale. The index date does not necessarily correspond to conception or delivery and may occur at different gestational stages. For example, if a case was first diagnosed with pregnancy on 1 January 2000, we would include it in the cohort from that date and begin follow‐up. The corresponding control members for this case would also have 1 January 2000, as their enrollment date. Importantly, controls were assigned the same index date as their matched exposed case, thereby aligning calendar time and minimizing bias from secular changes in healthcare access, screening practices, and diagnostic tendencies across the 1996–2013 study period. After excluding participants with a history of teenage pregnancy and those with any prior diagnosis of MPD before enrollment, the control group was randomly selected based on age, family income, residence location, and individual psychiatric comorbidity for matching (1:4). The urbanization level of residence (levels 1–4, from most to least urbanized) was assessed as a proxy for healthcare availability in Taiwan.^22^ A previous NHIRD study reported that individuals residing in areas with lower urbanization levels tended to utilize outpatient services less than those residing in areas with higher levels of urbanization.^23^ From enrollment to 31 December 2013, MPDs were diagnosed by board‐certified psychiatrists at least twice, which might improve the accuracy of the diagnoses,^24^ based on ICD‐9‐CM codes: 295 for schizophrenia; 296 (excluding 296.2*x*, 296.3*x*, 296.9*x*, and 296.82) for bipolar disorder; and 296.2, 296.3, 300.4, and 311 for major depressive disorder. Psychotic and affective disorder codes have been validated in the NHIRD, supporting the robustness of using such coding definitions.^25^ Because outcomes were identified from psychiatric service claims, findings may preferentially capture treated clinically significant cases and underestimate untreated/community cases; differential healthcare contact after adolescent pregnancy may also affect detection. In terms of event definition, schizophrenia and bipolar disorder take precedence over major depressive disorder. For example, if an individual is initially diagnosed with major depressive disorder and later, 2 years afterward, the diagnosis is changed to bipolar disorder, the event is defined as bipolar disorder. This hierarchy was applied to reduce outcome misclassification and double counting in claims data, where early depressive presentations may later be reclassified as bipolar disorder or schizophrenia after longitudinal clinical observation. As a result, our major depressive disorder incidence likely reflects cases that remain unipolar during follow‐up and may be conservatively estimated, because some initially coded depressive episodes that subsequently convert to bipolar disorder/schizophrenia are counted under the later, higher‐priority diagnosis. Participants were followed from the index date until the first occurrence of the outcome, withdrawal from the insurance program, death, or 31 December 2013, whichever came first. In addition, parental MPDs were also assessed. Individual psychiatric comorbidities, including attention‐deficit/hyperactivity disorder, alcohol use disorder, and substance use disorder, were evaluated. We also examined the Charlson Comorbidity Index (CCI) and all‐cause clinical visits for the two groups. The CCI, consisting of 17 diseases categories, was assessed to determine the systemic health conditions of all enrolled subjects^26^ (Supplement S1). Finally, to reduce selection bias, all‐cause clinical visits were assessed between the two groups.

### Ethics Consideration

The research methodology was approved by the Institutional Review Board of Taipei Veterans General Hospital (2018‐07‐16AC), and informed consent was not required since the study solely used de‐identified data and involved no human contact.

### Statistical analysis

Between‐group comparisons were performed using Pearson's chi‐squared tests for categorical variables and *F*‐tests for continuous variables. Kaplan–Meier survival analyses with log‐rank tests were conducted to compare the cumulative incidence of subsequent schizophrenia, bipolar disorder, and major depressive disorder between the adolescent pregnancy group and the control group. Cox proportional hazards regression models were used to estimate hazard ratios (HRs) and 95% confidence intervals (CIs) for each outcome, adjusting for demographic factors (age, sex, family income, and level of urbanization), individual psychiatric comorbidities, parental history of MPDs, CCI scores, and all‐cause clinical visits. In addition, stratified analyses were conducted to investigate the risks of MPDs according to the age at pregnancy (<16 vs ≥16 years). We selected 16 years as the *a priori* cutoff for stratified analyses because, during the study period (1996–2013), Taiwan's Civil Code set the minimum legal marriage age at 16 for females (18 for males), and the legal framework for sexual autonomy in Taiwan is commonly operationalized around age 16 in criminal‐law provisions regarding sexual activity with minors.

Notably, Taiwan later amended the Civil Code to unify the minimum marriage age to 18 years for both sexes, effective 1 January 2023; however, this change occurred after our study period and thus does not affect the current analyses. Sensitivity analyses were performed by excluding outcomes occurring within the first 1, 2, and 3 years of follow‐up. We illustrated the relationship between pregnancy age group and the timing of subsequent major depressive disorder onset. A two‐tailed p‐value of less than 0.05 was considered statistically significant. Data processing and statistical analyses were performed using SPSS version 17 and SAS version 9.1, developed by SAS Institute Inc., Cary, NC.

## Results

We identified 29,974 girls with teenage pregnancy and 119,896 age‐, family income‐, residence location‐, and individual psychiatric comorbidity‐matched controls (Table 1). The mean age of both the adolescent pregnancy group and the control group was 18.16 years. In the case group, 9.1% of adolescents became pregnant before the age of 16 years, while 90.9% became pregnant at or after 16 years. The adolescent pregnancy group had a higher prevalence of parental schizophrenia (0.2% vs 0.1%, p = 0.007), bipolar disorder (0.1% vs 0.1%, *P* = 0.016), and major depressive disorder (1.2% vs 0.9%, *P* < 0.001) compared to the control group. The adolescent pregnancy group also had a higher mean CCI score (0.69 vs 0.54, *P* < 0.001) and more frequent all‐cause clinical visits (times per year: 6.86 vs 4.59, *P* < 0.001) than the control group.

**Table 1:** Demographic and clinical characteristics between groups

Characteristics	Controls, *n* = 119,896	Adolescent pregnancy, *n* = 29,974	*P*‐value
Age (years, SD)	18.16 (1.55)	18.16 (1.50)	0.848
<16 years	10,908 (9.1)	2727 (9.1)	
≥16 years	108,988 (90.9)	27,247 (90.9)	
Female (*n*, %)	119,896 (100.0)	29,974 (100.0)	>0.999
Individual psychiatric comorbidities prior to any major psychiatric disorder			
ADHD	180 (0.2)	45 (0.2)	0.993
Alcohol use disorder	1016 (0.8)	254 (0.8)	0.997
Substance use disorder	1360 (1.1)	340 (1.1)	0.998
Parental major psychiatric disorder (*n*, %)			
Schizophrenia	116 (0.1)	47 (0.2)	0.007
Bipolar disorder	107 (0.1)	42 (0.1)	0.016
Major depressive disorder	1134 (0.9)	355 (1.2)	<0.001
CCI scores (SD)	0.54 (0.83)	0.69 (0.97)	<0.001
Level of urbanization (*n*, %)			>0.999
1 (most urbanized)	13,256 (11.1)	3314 (11.1)	
2	30,904 (25.8)	7726 (25.8)	
3	12,172 (10.1)	3043 (10.1)	
4	12,860 (10.7)	3215 (10.7)	
5 (most rural)	50,704 (42.3)	12,676 (42.3)	
Income‐related insured amount (*n*, %)			>0.999
≤19,100 NTD/month	38,736 (32.3)	9684 (32.3)	
19,001–42,000 NTD/month	56,600 (47.2)	14,150 (47.2)	
>42,000 NTD/month	24,560 (20.5)	6140 (20.5)	
All‐cause clinical visits (times per year, SD)	4.59 (3.78)	6.86 (4.38)	<0.001

CCI, Charlson comorbidity index; NTD, New Taiwan dollars; SD, standard deviation.

**Fig. 1:**
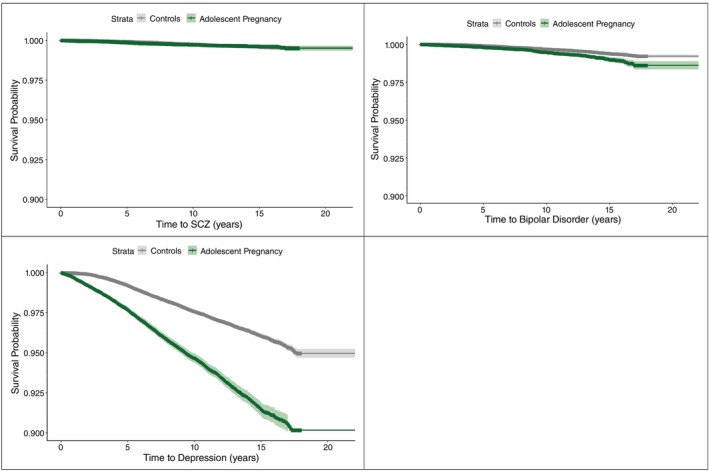
Survival curves of developing major psychiatric disorders between groups SCZ, schizophrenia.

**Fig. 2:**
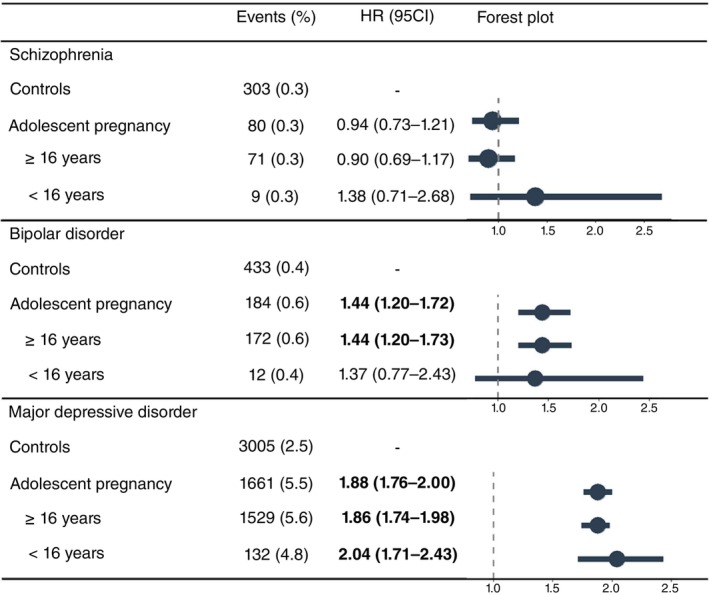
Risk of major psychiatric disorder between groups. CCI, Charlson comorbidity index; CI, confidence interval; HR, hazard ratio. Bold type indicated statistical significance (*P* < 0.05). Models were adjusted for age, income, residence, individual psychiatric comorbidities, parental major psychiatric disorders, Charlson Comorbidity Index (CCI) scores, and all‐cause clinical visit frequency.

The adolescent pregnancy group had a significantly higher proportion of subsequent bipolar disorder (0.6% vs 0.4%, *P* < 0.001), major depressive disorder (5.5% vs 2.5%, *P* < 0.001), and any MPD (6.4% vs 3.1%, *P* < 0.001) than the control group, but not schizophrenia (0.3% vs 0.3%, *P* = 0.7). The Kaplan–Meier survival curves are presented in Fig. 1. After adjustment for age, income, residence, individual psychiatric comorbidities, parental MPDs, CCI scores, and all‐cause clinical visits, individuals with adolescent pregnancy had a higher risk of developing bipolar disorder (HR = 1.44, 95% CI = 1.20 to 1.72) and major depressive disorder (HR = 1.88, 95% CI = 1.76 to 2.00; Table 2). However, the adolescent pregnancy group did not have a different risk of schizophrenia (HR = 0.94, 95% CI = 0.73 to 1.21) compared to the control group (Fig. 2).

**Table 2:** Proportion of the major psychiatric disorder between groups

Characteristic	Controls, *n* = 119,896^†^	Adolescent pregnancy, *n* = 29,974^†^	*P*‐value^‡^
Schizophrenia	303 (0.3%)	80 (0.3%)	0.7
Major depressive disorder	3005 (2.5%)	1661 (5.5%)	<0.001
Bipolar disorder	433 (0.4%)	184 (0.6%)	<0.001
Any major psychiatric disorder	3741 (3.1%)	1925 (6.4%)	<0.001

^†^ *n* (%).

^‡^ Pearson's chi‐squared test.

Stratified by pregnancy age (Fig. 2), only adolescent girls who became pregnant after the age of 16 years had a significantly higher risk of developing bipolar disorder (HR = 1.44, 95% CI = 1.20 to 1.73), while those who became pregnant before 16 years did not show a statistically significant increase in risk. The adolescent pregnancy group, regardless of whether they became pregnant before or after the age of 16 years, had a higher risk of developing major depressive disorder (≥16 years, HR = 1.86, 95% CI = 1.74 to 1.98; <16 years, HR = 2.04, 95% CI = 1.71 to 2.43). The association between pregnancy age and major depressive disorder, bipolar disorder, and schizophrenia onset are shown in Supplements S2–S4. Across the four groups (≥18, 16–18, 14–16, and <14 years old), there is a decreasing trend in pregnancy age corresponding with an earlier age of major depressive disorder onset (Supplement S2). Specifically, individuals with pregnancy age ≥18 had the highest mean age of major depressive disorder onset (mean = 26.45, SD = 4.05), while those with pregnancy age <14 had the lowest (mean = 21.35, SD = 3.45). Median values mirrored this trend, decreasing from 25.90 [Q1 = 23.20, Q3 = 29.42] in the ≥18 group to 22.20 [Q1 = 19.39, Q3 = 23.22] in the <14 group. Age at bipolar disorder (Supplement S3) onset also tended to decrease with younger pregnancy age. The mean onset age declined from 27.41 years (SD = 4.19) in the ≥18 group to 23.89 years (SD = 5.15) in the <14 group, although the <14 group had a very small sample size. A similar decreasing trend was observed for schizophrenia onset across pregnancy age groups (Supplement S4). The mean onset age fell from 26.25 years (SD = 4.04) in the ≥18 group to 20.57 years (SD = 3.30) in the <14 group, and the median values showed a consistent pattern.

In the sensitivity analyses excluding early‐onset MPDs, the findings remained robust (Table 3). After excluding MPD cases occurring within the first 3 years after the pregnancy index date, the adolescent pregnancy group still had higher risks of bipolar disorder (HR = 1.27, 95% CI = 1.05 to 1.54) and major depressive disorder (HR = 1.61, 95% CI = 1.50 to 1.72) compared to the control group.

**Table 3:** Sensitivity analyses excluding early‐onset severe mental disorders after cohort entry

	Excluding <1 year	Excluding <2 years	Excluding <3 years
Schizophrenia			
Control group	302 (0.3)	287 (0.2)	262 (0.2)
Adolescent pregnancy group	74 (0.3)	68 (0.2)	62 (0.2)
Hazard ratios (95% CI)	0.85 (0.65–1.10)	0.82 (0.62–1.07)	0.83 (0.63–1.11)
Bipolar disorder			
Control group	432 (0.4)	422 (0.4)	403 (0.3)
Adolescent pregnancy group	176 (0.6)	164 (0.5)	156 (0.5)
Hazard ratios (95% CI)	1.35 (1.12–1.61)	1.28 (1.06–1.54)	1.27 (1.05–1.54)
Major depressive disorder			
Control group	2,979 (2.5)	2,906 (2.4)	2,712 (2.3)
Adolescent pregnancy group	1,566 (5.2)	1,437 (4.8)	1,315 (4.4)
Hazard ratios (95% CI)	1.75 (1.64–1.87)	1.64 (1.57–1.75)	1.61 (1.50–1.72)

CI, confidence interval.

## Discussion

In this longitudinal nationwide cohort study with 29,974 adolescent pregnant girls, we found that teenage pregnancy was associated with increased risks of subsequent diagnoses of bipolar disorder and major depressive disorder, but not schizophrenia. Stratified by pregnancy age, the increased risk of subsequent bipolar disorder was observed in individuals who became pregnant after the age of 16 years, but not in the subgroup who became pregnant before 16 years. The increased risk of major depressive disorder was observed in both groups – those who became pregnant before and after the age of 16. Even after excluding short‐term onset MPDs, the higher long‐term risks of subsequent bipolar disorder and major depressive disorder in the adolescent pregnancy group remained apparent. Given the observational nature of this nationwide cohort study, these associations should be interpreted as non‐causal and may reflect residual confounding despite adjustment.

Previous studies have shown that individuals with postpartum psychosis have a relatively higher likelihood of being subsequently diagnosed with bipolar disorder than with schizophrenia.^13^, ^15^ This has been associated with factors such as hormonal changes (including estrogen, progesterone, prolactin, follicle‐stimulating hormone, and luteinizing hormone), alterations in the immune system, sleep deprivation, genetic factors, and various psychosocial factors.^13^, ^27^, ^28^, ^29^, ^30^, ^31^ However, our study (as shown in the Kaplan–Meier curve) indicates that not only immediately postpartum but also in the long term, adolescent pregnancy is associated with a higher likelihood of bipolar disorder. The incidence of bipolar disorder in the case group continues to significantly increase over time compared to the control group. This may suggest that it is not only the physiological changes during pregnancy but also the long‐term psychosocial stress following childbirth that can influence the onset of bipolar disorder.^32^ (Because the index date reflects the first claims‐based ascertainment of pregnancy rather than the delivery date, postpartum timing is discussed conceptually and cannot be treated as an exact clinical time point in our data.) Another possible explanation is that individuals with bipolar disorder may already exhibit certain traits (such as mood lability, recklessness, or high energy), or even subthreshold manic episodes, prior to their first mood episode, which could make them more prone to teenage pregnancy.^11^ Although the increased risk of bipolar disorder was not statistically significant in the early adolescent pregnancy subgroup (<16 years), the relatively small sample size limited the statistical power of the analysis – especially considering that the total number of cases in this group was only one‐tenth that of the ≥16 years group, with only 12 events observed. Beyond limited power, the age‐specific pattern may reflect differences in developmental stage and psychosocial context, as pregnancies occurring at ≥16 years may more often coincide with late‐adolescent transitions (e.g. greater autonomy, educational/vocational disruption, and role/relationship stress) that could amplify longer‐term vulnerability to bipolar disorder. Additionally, pregnancy‐related factors that may vary by age at pregnancy (e.g. pregnancy outcomes/complications and postpartum social support) were not captured in claims data and could contribute to heterogeneity in subsequent bipolar risk.

Regarding schizophrenia, despite genetic evidence suggesting shared causal factors between schizophrenia and bipolar disorder – including in cases of postpartum psychosis – we did not observe a similarly increased risk of schizophrenia following teenage pregnancy, as we did with bipolar disorder.^29^, ^31^, ^33^ The null association with schizophrenia (HR = 0.94) may indicate that the impact of adolescent pregnancy is more specific to affective disorders. One possible explanation is etiologic: although psychosocial stress is relevant to psychosis risk, schizophrenia has a stronger genetic and neurodevelopmental basis and may be less susceptible to the long‐term psychosocial sequelae of adolescent motherhood than bipolar disorder or major depressive disorder.^34^ This pattern is also broadly consistent with peripartum literature in which severe postpartum episodes often align more closely with bipolar spectrum illness, even though overlaps with schizophrenia liability exist.^35^ Finally, the point estimate slightly below 1.0 should be interpreted cautiously and may reflect a true null effect, limited power, or selection mechanisms (e.g. premorbid neurodevelopmental liability reducing the likelihood of pregnancy or exclusion due to pre‐index diagnosis), rather than a protective effect.

A recent study by Munk‐Olsen *et al*. found no clear evidence that depression occurring postpartum is genetically distinct from depression occurring outside the postpartum period, suggesting substantial shared genetic liability.^36^ However, this overlap does not imply equivalence, as peripartum depression is heterogeneous and may include hormone‐sensitive subtypes; emerging epigenetic biomarker work has reported that postpartum depression signatures can also identify other hormonally related depressions such as premenstrual dysphoric disorder and post‐menopausal depression.^37^ Our findings were consistent with previous studies – girls with a history of teenage pregnancy have a higher risk of developing major depressive disorder later in life. An Australian study with 4,262 female participants found that women who had their first child as teenagers are at significantly higher risk of poor long‐term mental health compared to those who gave birth at age 25 or older.^38^ Another large British cohort study reported that teenage motherhood was linked to an increased risk of depression and anxiety morbidity in adulthood.^39^ Socioeconomic disadvantage, social stigma, and exposure to adverse life events are significant factors that can exacerbate the challenges of motherhood and have enduring impacts on the mental health of young mothers.^40^ On the other hand, several risk factors are common to both teenage pregnancy and major depressive disorder, such as low economic status, childhood abuse, substance use, parental mental disorders, neglectful parenting styles, and single‐parent families.^4^, ^41^ Because parental history of MPDs reflects genetic and family environmental liability, we adjusted for it as a covariate in the models. The persistence of the association after this adjustment indicates that adolescent pregnancy may contribute additional risk beyond familial predisposition. Even though we have adjusted for many risk factors to the best of our ability, some risk factors – particularly key psychosocial and developmental factors not captured in claims data, such as childhood adversity/trauma, family dysfunction, educational disruption, and social support – cannot be captured in our study. Girls with these risk factors might have a higher likelihood of experiencing teenage pregnancy and major depressive disorder. However, those who were already diagnosed with major depressive disorder have been excluded from our study, while those with latent depression have been retained.

In the sensitivity analyses, we excluded cases of MPD that occurred within 1, 2, and 3 years after pregnancy to account for the potential influence of postpartum effects. Because follow‐up was anchored at the pregnancy index date (first claims‐based pregnancy ascertainment), the 1‐, 2‐, and 3‐year exclusion windows were defined as time since this index date. Assuming 40 weeks of gestation, a 1‐year window corresponds to approximately 2 months postpartum (with longer windows extending further beyond delivery). The 2‐ and 3‐year exclusion windows allow for an adjustment period for the mothers in terms of care burden, new life, and new roles. Even so, we still observed that the long‐term risk of bipolar disorder and major depressive disorder remained higher in the exposed group compared to the control group, even after 3 years. This suggests that the contributing factors are not limited to postpartum hormonal changes or the challenges associated with the arrival of a newborn. In the figure showing the relationship between age at pregnancy and depression onset, we can also observe that, on average, major depressive disorder is diagnosed around 7–8 years after an adolescent pregnancy. This pattern is consistently observed across all four age groups (≥18, 16–18, 14–16, and <14 years old). Of course, in our study, the 7–8 year interval refers to the time of major depression diagnosis, not the actual onset. It is possible that depressive symptoms had been present for some time before the individual sought medical help. Previous studies have reported that individuals often delay seeking treatment for mood disorders during the postpartum period or while caring for an infant. Possible reasons include practical barriers (such as financial constraints and lack of childcare), stigma, and a lack of awareness.^42^ A mother's mood disorders are closely linked to the child's development (including cognitive, motor, emotional, and language development),^43^, ^44^ as well as their safety (hospitalization and death).^45^ Early detection and intervention for maternal mood disorders become even more crucial.

### Limitation

Nevertheless, our study still had several limitations. First, the study covered a long accrual period (1996–2013), during which mental‐health awareness, stigma, healthcare utilization, and diagnostic practice likely changed. Although cases and matched controls were aligned on the same index date to reduce calendar‐time confounding, residual ascertainment bias remains possible if adolescents with pregnancy had differential contact with health services, leading to increased opportunities for diagnosis over time. Accordingly, the observed effect estimates may not directly reflect the magnitude of associations under current mental‐health awareness and service patterns, although the direction of associations may remain informative for long‐term surveillance. Moreover, the adolescent pregnancy group had substantially higher all‐cause clinical visits, which may increase the likelihood of psychiatric detection and diagnostic recording through more frequent healthcare contact. Although we adjusted for visit frequency, residual detection bias may persist because visit counts may not fully capture differential diagnostic opportunity or clinical intensity; if present, this would more likely inflate the estimated effect sizes (magnitude) than reverse the direction of the associations. Second, psychiatric outcomes were identified from administrative ICD codes rather than structured interviews; thus, changes in diagnostic practices and service utilization may influence recorded incidence, and misclassification cannot be fully excluded despite requiring repeated psychiatrist diagnoses. Third, important psychosocial and developmental factors (e.g. early‐life adversity, family dysfunction, trauma, social support, and lifestyle behaviors) were not available in the database and therefore could not be adjusted for, leaving the possibility of residual confounding. Fourth, because our follow‐up period is limited (1996–2013), it is possible that some events or diagnostic shifts (e.g. major depressive disorder evolving into bipolar disorder) occurred after the follow‐up period, which may lead to underestimation of true lifetime outcomes and diagnostic transitions occurring after the observation window. Fifth, our exposure definition captured adolescent pregnancy using a composite of pregnancy‐related diagnostic and procedure codes and therefore combined pregnancies ending in live birth, spontaneous abortion, and induced abortion. These outcomes may have different biological and psychosocial implications (e.g. postpartum caregiving burden after live birth *versus* stress, grief, stigma, or medical complications related to pregnancy loss or termination). We did not conduct stratified analyses by pregnancy outcome because claims data do not always permit reliable, mutually exclusive outcome classification for each pregnancy, particularly for early losses or when care occurs across institutions, and subdividing the cohort would markedly reduce event counts for severe mood disorders, yielding unstable estimates. Accordingly, our findings should be interpreted as an average association across heterogeneous pregnancy outcomes, and future work using datasets with more detailed obstetric information is warranted. Lastly, generalizability should be considered. Taiwan has near‐universal health insurance coverage and relatively accessible maternal and psychiatric services, which may influence help‐seeking, diagnostic capture, and the availability of postpartum and social support compared with settings with more limited coverage. Therefore, while the direction of the associations may be informative, the magnitude of the observed risks may not directly generalize to countries with different social welfare policies, reproductive health services, or cultural norms surrounding adolescent pregnancy and mental illness. Cross‐national replication across diverse healthcare and sociocultural contexts is warranted.

## Conclusion

After adjusting for available confounders, adolescent pregnancy was associated with increased subsequent risks of bipolar disorder and major depressive disorder beyond the immediate postpartum period. Although mechanisms and clinical phenotyping were not available in this claims‐based cohort, these nationwide data provide population‐level evidence to support sustained, long‐term mental health surveillance and timely access to psychiatric care for adolescent mothers, in addition to socioeconomic support. Nevertheless, replication using more recent data and in other settings is warranted.

## Author contributions

T‐W.H. and J‐R.L. drafted the first version of the manuscript. M‐H.C., J‐W.H., and C‐S.L. designed the study. C‐S.L. and M‐H.C. analyzed the data. S‐J.T., Y‐M.B., T‐P.S., J‐W.H., and T‐J.C. performed the literature search and reviewed the manuscript. C‐S.L. has accessed and verified the data. C‐S.L. and M‐H.C. were responsible for the decision to submit the manuscript. All authors contributed substantially to the manuscript and approved the final manuscript for submission. All authors are responsible for the integrity, accuracy, and presentation of the data.

## Funding

The study was supported by a grant from Taipei Veterans General Hospital (V113C‐039, V113C‐011, V113C‐010, V114C‐089, V114C‐064, and V114C‐217); Yen Tjing Ling Medical Foundation (CI‐113‐32, CI‐113‐30, and CI‐114‐35); Ministry of Science and Technology, Taiwan (MOST111‐2314‐B‐075‐014‐MY2, MOST 111‐2314‐B‐075‐013, and NSTC113‐2314‐B‐075‐042); Taipei, Taichung, Kaohsiung Veterans General Hospital, Tri‐Service General Hospital, Academia Sinica Joint Research Program (VTA112‐V1‐6‐1 and VTA114‐V1‐4‐1); Veterans General Hospitals and University System of Taiwan Joint Research Program (VGHUST112‐G1‐8‐1 and VGHUST114‐G1‐9‐1); and Cheng Hsin General Hospital (CY11402‐1 and CY11402‐2). The funding source had no role in any process of our study.

## Disclosure statement

The authors have no conflicts of interest to declare.

## Supporting information


**Supplement S1.** ICD‐9 codes for CCI score.
**Supplement S2**. Depression onset by different pregnant age group.
**Supplement S3**. Bipolar disorder onset by different pregnant age group.
**Supplement S4**. Schizophrenia onset by different pregnant age group.

## Data Availability

The NHIRD was released and audited by the Department of Health and the Bureau of the NHI Program for the purpose of scientific research (https://www.apre.mohw.gov.tw/). The NHIRD can be accessed through a formal application regulated by the Health and Welfare Data Science Center of the Ministry of Health and Welfare, Taiwan.
