# Supplementary material for: Risk of major psychiatric disorder after adolescent pregnancy: A nationwide cohort study of 149,870 girls
Source: Psychiatry Clin Neurosci. 2026 Mar 26;80(6):519–26. doi: 10.1111/pcn.70054 (PMC13244586; doi:10.1111/pcn.70054)
Supplement: Supplementary file 1 — Supplement S1. ICD‐9 codes for CCI score. Supplement S2. Depression onset by different pregnant age group. Supplement S3. Bipolar disorder onset by different pregnant age group. Supplement S4. Schizophrenia onset by different pregnant age group. [file PCN-80-519-s001.docx]

Supplement 1. ICD-9 codes for CCI score

|  |  |
| --- | --- |
| Age | <50, 50-59, 60-69, 70-79, ≧80 |
| Myocardial infarction | 410.x, 412.x |
| Congestive heart failure | 398.91, 402.01, 402.11, 402.91, 404.01, 404.03, 404.11, 404.13, 404.91, 404.93, 425.4–425.9, 428.x |
| Peripheral vascular disease | 093.0, 437.3, 440.x, 441.x, 443.1–443.9, 447.1, 557.1, 557.9, V43.4 |
| Cerebrovascular disease | 362.34, 430.x–438.x |
| Dementia | 290.x, 294.1, 331.2 |
| Chronic pulmonary disease | 416.8, 416.9, 490.x–505.x, 506.4, 508.1, 508.8 |
| Connective tissue disease | 710.0, 710.1, 710.4, 714.0–714.2, 714.81, 725.x |
| Peptic ulcer disease | 531.x–534.x |
| Mild liver disease | 571.2, 571.4–571.6, 573.3, 573.4, 573.8, 573.9, V42.7 |
| Moderate or severe liver disease | 456.0–456.2, 572.2–572.8 |
| Diabetes without chronic complication | 250.0–250.3, 250.7 |
| Diabetes with chronic complication | 250.4–250.6 |
| Hemiplegia/paraplegia | 342.x, 343.x, 344.1 |
| Renal disease | 403.01, 403.11, 403.91, 404.02, 404.03, 404.12, 404.13, 404.92, 404.93, 582.x, 583.0–583.7, 585.x, 586.x, 588.x, V42.0, V45.1, V56.x |
| Any malignancy | 140.x–172.x, 174.x–195.8, 200.x–208.x |
| Metastatic solid tumor | 196.x–199.x |
| AIDS/HIV | 042.x–044.x |

Supplement 2. Depression onset by different pregnant age group

| 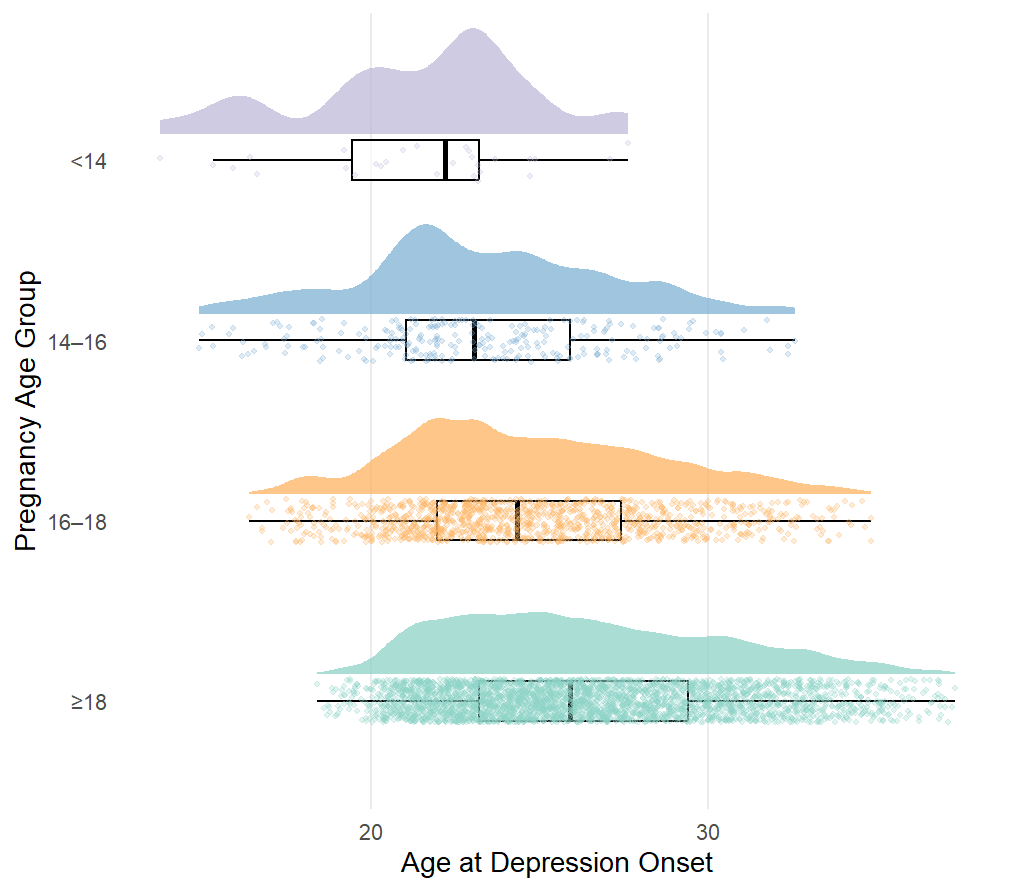 | 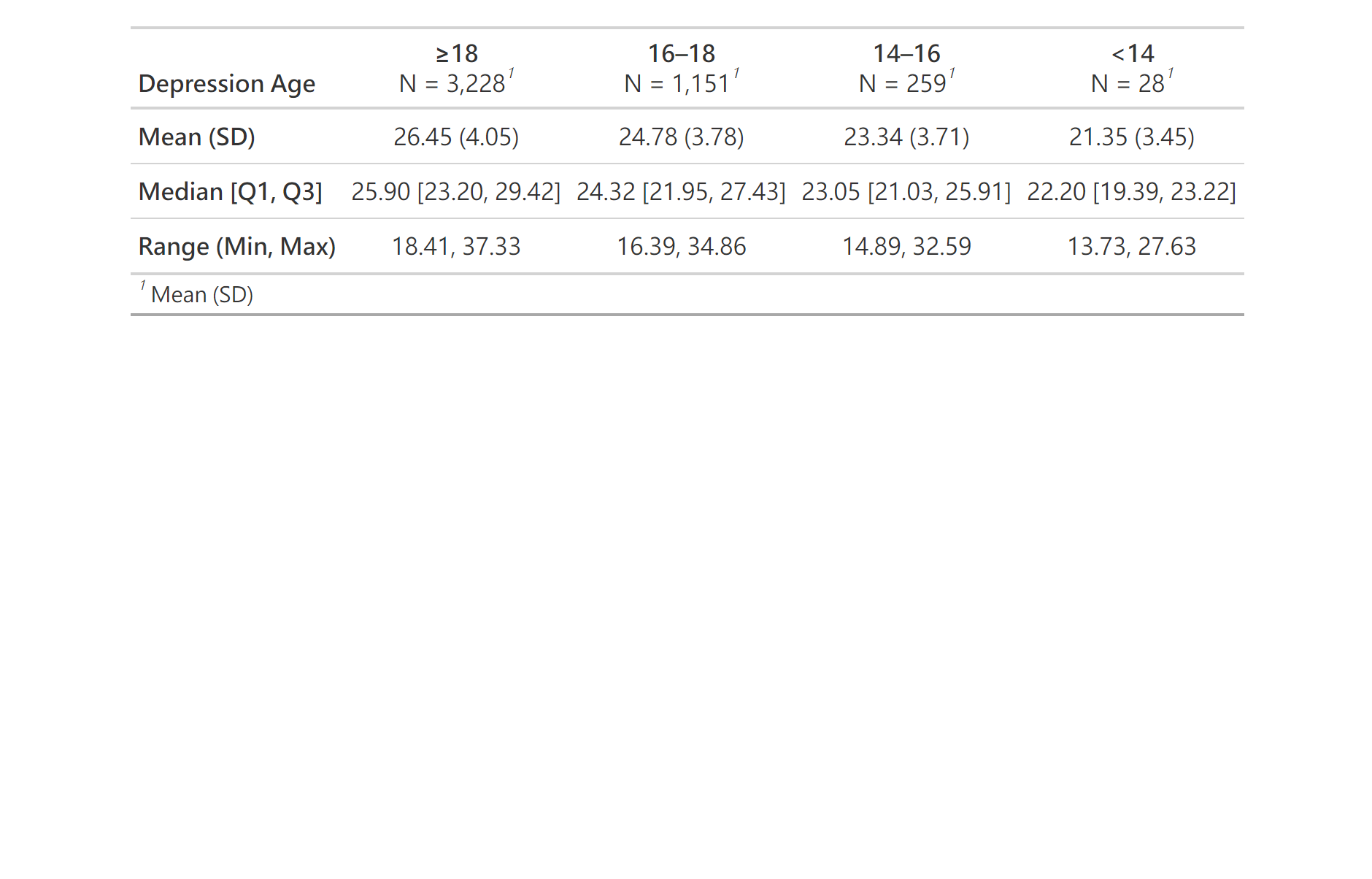 |
| --- | --- |

Supplement 3. Bipolar disorder onset by different pregnant age group

| 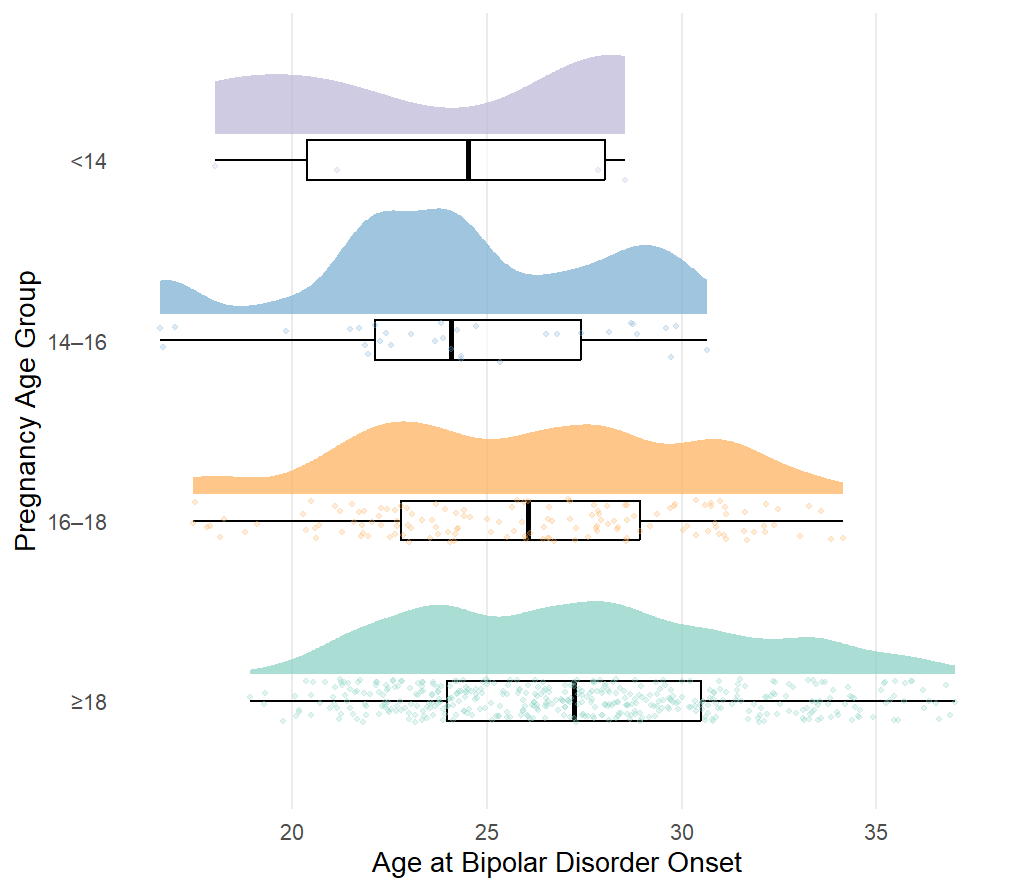 | 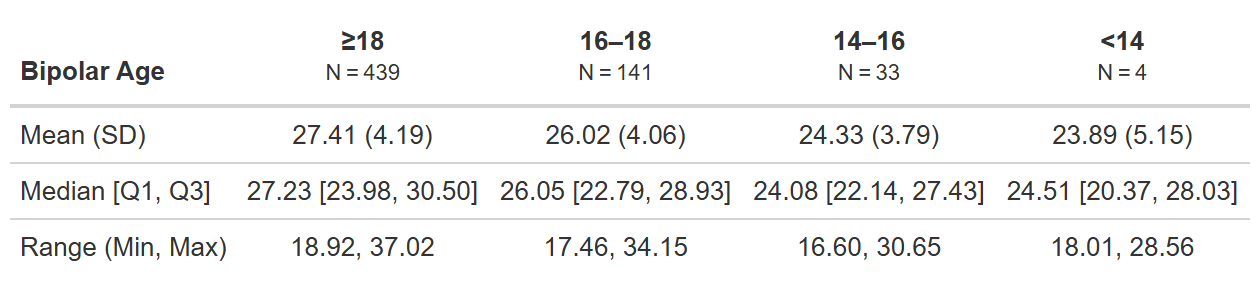 |
| --- | --- |

Supplement 4. Schizophrenia onset by different pregnant age group

| 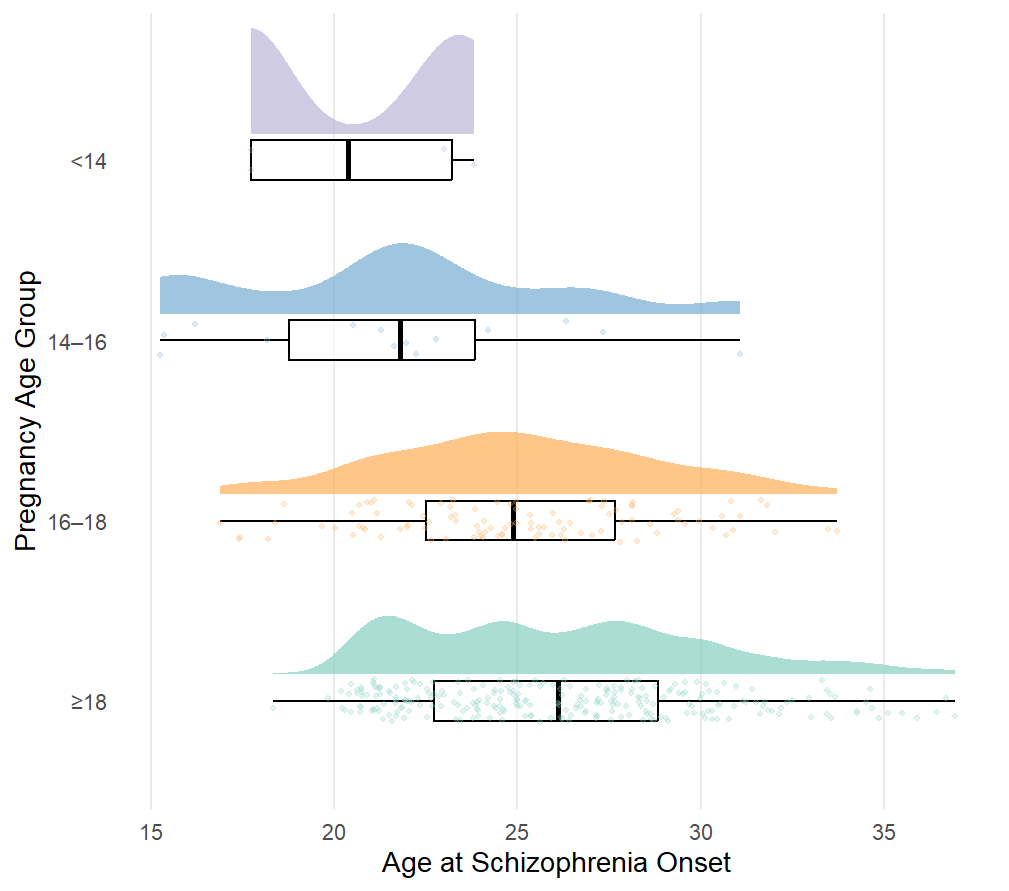 | 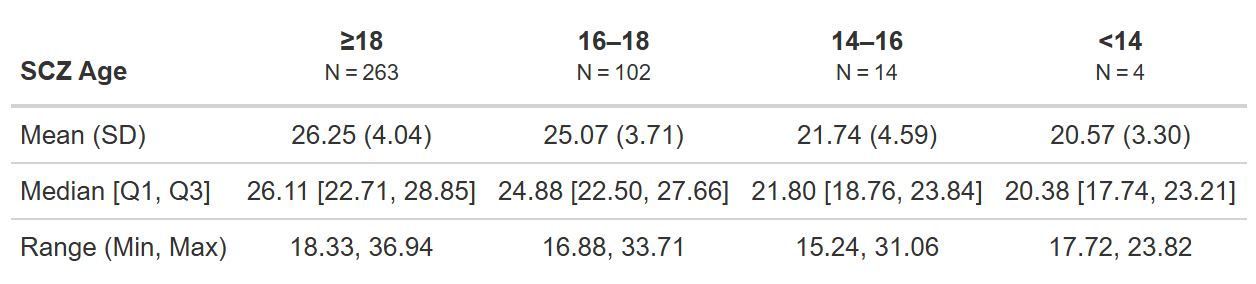 |
| --- | --- |
